# Health Physician and the Report of Deaths

**Published:** 1864-03

**Authors:** 


### HEALTH PHYSICIAN AND THE REPORT OF DEATHS.

We notice that our friend "Medicus," in his remarks upon intus-susception, and under his own signature, concludes that the present Health Physician is not aware that there is a Medical Journal published in Buffalo, or is indifferent to the interests of the profession, since he does not furnish it with his monthly report of deaths; also, he thinks possibly the Editor is not anxious to give it a place, or willing to make sufficient effort to obtain it.

We do not propose to speak for the Health Physician. Our pages are open, he can speak for himself. The causes of death in Buffalo are estimated by physicians in about one-half of the number as usually reported while the remainder is made up by Friends, or Undertakers, and Quacks. Physicians attach no great importance to the report, since convulsions, teething, anæmia, disease of the brain, heart, lungs, spine, kidney, etc., etc., are the headings under which are arranged the causes of death, even by physicians, to say nothing of other sources from which the Health Physician makes out his monthly report. If the Health Physician should report ten cases of intus susception in a year, it would never occur to the Editor of this *Journal* that a single case had actually occurred, unless he should fill up by whom certified, and the number verified by *post-mortem* examination. It requires no argument to show that the report of deaths, as furnished, is the merest farce in the world, and though the Editor is ready to publish and to make all reasonable effort to obtain it, still he would not like to have any one infer that he regards the record of the causes of death of any value whatever. It would be well to place in the *Journal* this report imperfect, as it must necessarily be, not that it would show cause of death, but it does show the number of deaths, where they occur, who certifies to them, between what ages, and from what country. The causes of death are unknown to the Health Physician, and all intelligent physicians, who bestow a moment's thought upon it, will discover that under our present system of reporting the causes of death the profession lose nothing if it is unpublished. It was for a time placed in the *Journal* as an original communication, and often occupied two or three pages. While we do not regard it worthy of space at great length, for the reasons we have assigned, still we shall feel under deep obligations to the Health Physician if he will furnish us his report for publication. We have no doubt the present efficient Health officer is most fully aware of the objections we have suggested, and regrets his inability to furnish more reliable statistics.

